# ColistinDose, a Mobile App for Determining Intravenous Dosage Regimens of Colistimethate in Critically Ill Adult Patients: Clinician-Centered Design and Development Study

**DOI:** 10.2196/20525

**Published:** 2020-12-16

**Authors:** Xueliang Hua, Chen Li, Jason M Pogue, Varun S Sharma, Ilias Karaiskos, Keith S Kaye, Brian T Tsuji, Phillip J Bergen, Yan Zhu, Jiangning Song, Jian Li

**Affiliations:** 1 Independent Researcher Santa Clara, CA United States; 2 Department of Biochemistry and Molecular Biology Monash University Melbourne Australia; 3 Infection and Immunity Program Biomedicine Discovery Institute Monash University Melbourne Australia; 4 Institute of Molecular Systems Biology Department of Biology ETH Zurich Zurich Switzerland; 5 Department of Clinical Pharmacy University of Michigan College of Pharmacy Ann Arbor, MI United States; 6 1st Internal Medicine and Infectious Diseases Department Hygeia Hospital Marousi Greece; 7 Department of Internal Medicine University of Michigan Medical School Ann Arbor, MI United States; 8 Laboratory for Antimicrobial Dynamics NYS Center of Excellence in Bioinformatics & Life Sciences Buffalo, NY United States; 9 School of Pharmacy and Pharmaceutical Sciences University at Buffalo Buffalo, NY United States; 10 Department of Microbiology Monash University Melbourne Australia; 11 Monash Centre for Data Science Faculty of Information Technology Monash University Melbourne Australia

**Keywords:** ColistinDose, colistimethate, colistin methanesulfonate, colistin, polymyxins, mobile app, renal function, renal replacement therapy, intermittent hemodialysis, sustained low-efficiency dialysis, continuous renal replacement therapy

## Abstract

**Background:**

Determining a suitable dose of intravenous colistimethate is challenging because of complicated pharmacokinetics, confusing terminology, and the potential for renal toxicity. Only recently have reliable pharmacokinetic/pharmacodynamic data and dosing recommendations for intravenous colistimethate become available.

**Objective:**

The aim of this work was to develop a clinician-friendly, easy-to-use mobile app incorporating up-to-date dosing recommendations for intravenous colistimethate in critically ill adult patients.

**Methods:**

Swift programming language and common libraries were used for the development of an app, ColistinDose, on the iPhone operating system (iOS; Apple Inc). The compatibility among different iOS versions and mobile devices was validated. Dosing calculations were based on equations developed in our recent population pharmacokinetic study. Recommended doses generated by the app were validated by comparison against doses calculated manually using the appropriate equations.

**Results:**

ColistinDose provides 3 major functionalities, namely (1) calculation of a loading dose, (2) calculation of a daily dose based on the renal function of the patient (including differing types of renal replacement therapies), and (3) retrieval of historical calculation results. It is freely available at the Apple App Store for iOS (version 9 and above). Calculated doses accurately reflected doses recommended in patients with varying degrees of renal function based on the published equations. ColistinDose performs calculations on a local mobile device (iPhone or iPad) without the need for an internet connection.

**Conclusions:**

With its user-friendly interface, ColistinDose provides an accurate and easy-to-use tool for clinicians to calculate dosage regimens of intravenous colistimethate in critically ill patients with varying degrees of renal function. It has significant potential to avoid the prescribing errors and patient safety issues that currently confound the clinical use of colistimethate, thereby optimizing patient treatment.

## Introduction

As the drug discovery pipeline for new antibiotics has dwindled [1,2], multidrug-resistant gram-negative pathogens have become a serious global health threat [3]. Colistin (ie, polymyxin E) has increasingly been used as a “last-line” therapy to treat infections caused by gram-negative “superbugs” unresponsive to other agents [4,5]. In clinical settings, colistin is most commonly available as an inactive prodrug, colistimethate (also known as colistin methanesulfonate [CMS]), for intravenous and inhalational administration [6]. Unfortunately, nephrotoxicity following intravenous administration of polymyxins (colistin or polymyxin B) can occur in up to 60% of patients and is the major dose-limiting factor [7-9]. Owing to earlier difficulties in determining concentrations of colistimethate and formed colistin, only relatively recently have studies reliably investigated the pharmacokinetics (PK) of colistimethate and formed colistin in patients [10-18]. These studies have revealed the extremely complicated PK of colistimethate and formed colistin, which in turn has made determining appropriate dosing regimens for intravenous colistimethate very challenging. The complex PK of both the prodrug and formed colistin in patients extend to those with different renal functions and those on different renal replacement therapies (RRT) given that the apparent clearance of colistin is dependent on renal function [10]. Adding to this complexity is that the dose units of colistimethate are expressed differently in different parts of the world, namely as either colistin base activity (CBA) or number of international units (IU) (approximately 33.3 mg of CBA=1 million IU=approximately 80 mg of CMS); these different expressions are known to have caused prescribing errors and patient safety issues, and substantially confound the clinical use of colistimethate [19]. Selecting an optimal dose of colistimethate in critically ill patients is thus a difficult process with serious consequences for both underdosing (treatment failure and development of resistance) and overdosing (toxicity).

Mobile devices have become commonplace in health care settings. Professional mobile apps have created paradigm shifts in modern medicine in a number of areas including information storage and access, patient management and monitoring, clinical decision making, and clinical practice transformation [20]. Given the role of colistimethate as one of the most important last-line therapies for multidrug-resistant gram-negative bacteria and the difficulties associated with accurately determining an appropriate dose across many patient groups (including those on RRT), a user-friendly smartphone app utilizing the latest clinical pharmacological findings to determine optimal, personalized dosing regimens of intravenous colistimethate in patients would provide valuable assistance to clinicians.

We recently published the most comprehensive population PK model to date with dosing recommendations based on data from a total of 214 critically ill patients that included 29 patients on different forms of RRT [11]. To the best of our knowledge, there are no apps and only two online tools available for the calculation of colistimethate dosage regimens: a colistin dosing calculator [21] and a colistin calculator [22]. Neither tool utilizes the latest population PK model [11] for calculating the dosage regimens of intravenous colistimethate. This study aimed to develop an app, ColistinDose, for the iPhone operating system (iOS; Apple Inc) to allow clinicians to accurately and conveniently determine the dosing regimens of intravenous colistimethate on a local iPhone or iPad. Our app provides clinicians with an easy-to-use tool for determining optimal dosing regimens of intravenous colistimethate in adult patients with varying degrees of renal function directly at the bedside.

## Methods

### Development of the ColistinDose Smartphone App

**Figure 1:**
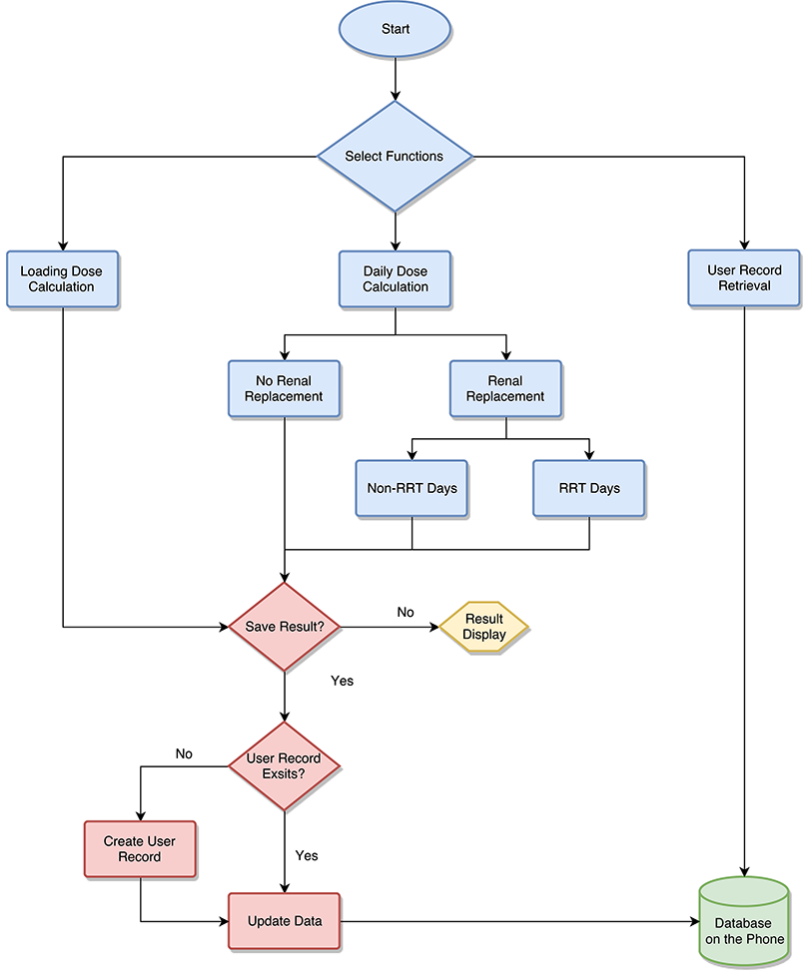
The workflow of ColistinDose. RRT: renal replacement therapy.

Xcode and Interface Builder (Apple Inc) were employed to build the app’s human interaction interfaces. Several open-source libraries (including Charts, Former, Persei, and Realm; please refer to the Multimedia Appendix 1 and the Acknowledgments section in the app for detailed information) were also utilized to implement relevant user interface elements, animation, and, in particular, local data storage. The overall design of the workflow of ColistinDose is shown in Figure 1. We employed the Swift programming language and common libraries for iOS to ensure that ColistinDose was functional on different screen sizes and versions of the operating system on iPhones and iPads.

### PK/Pharmacodynamics (PD) Models of Intravenous Colistimethate in Patients for ColistinDose

In 2017, we published dosing suggestions of intravenous colistimethate based on the largest population PK study conducted to date [11], including 214 critically ill patients (composed of 105 patients from an interim analysis [10] and 109 patients from an additional study site in Greece). In particular, the 2017 study included a larger number of patients with low creatinine clearances (CrCls), including 16 patients on hemodialysis, 4 on sustained low-efficiency dialysis (SLED), 7 on continuous veno-venous hemodialysis (CVVHD), and 2 on continuous veno-venous hemofiltration (CVVH); these additional patients receiving RRT improved the ability to predict doses for these patients. The structural model from the interim analysis provided the starting point for further analysis, with all relevant covariates retested. After the new population PK analysis, the final model remained similar to the earlier model. For the calculation of the recommended loading dose (all patients), our final algorithm used ideal body weight (IBW) in all cases rather than the lower of actual body weight or IBW as per the interim analysis. The algorithm-derived maintenance (daily) dosing recommendations for patients with a CrCl >30 mL/min remained very similar to those proposed in the interim analysis. However, for patients with a CrCl <30 mL/min (including patients prescribed intermittent hemodialysis [IHD] who were on a nondialysis day), the new recommendations were approximately 100% higher than those suggested in the interim analysis and also substantially higher than the doses currently approved by the Food and Drug Administration [23]. By utilizing the most recent equations, ColistinDose provides the most accurate dosing recommendations, especially for patients with low CrCls.

### Calculations of Dosage Regimens Using ColistinDose

Calculations were based on the equations for the loading dose (all patients) and daily dose (for patients not receiving RRT and for patients on various types of RRT) developed in our recent population PK study [11]. Patients’ IBW (kg) was calculated using the following equation: IBW = 50 kg (45.5 kg for females) + 2.3 × number of inches over 5 feet (ie, 60 inches); CrCl (mL/min) was calculated using the Cockcroft-Gault equation [24]. Three major functionalities were implemented in ColistinDose, namely calculations of the loading dose and maintenance daily dose, plus retrieval of historical records saved on the device. Recommended doses generated by ColistinDose were validated by comparison against doses calculated manually using the appropriate equations from the population PK study [11].

## Results

### The ColistinDose App

**Figure 2:**
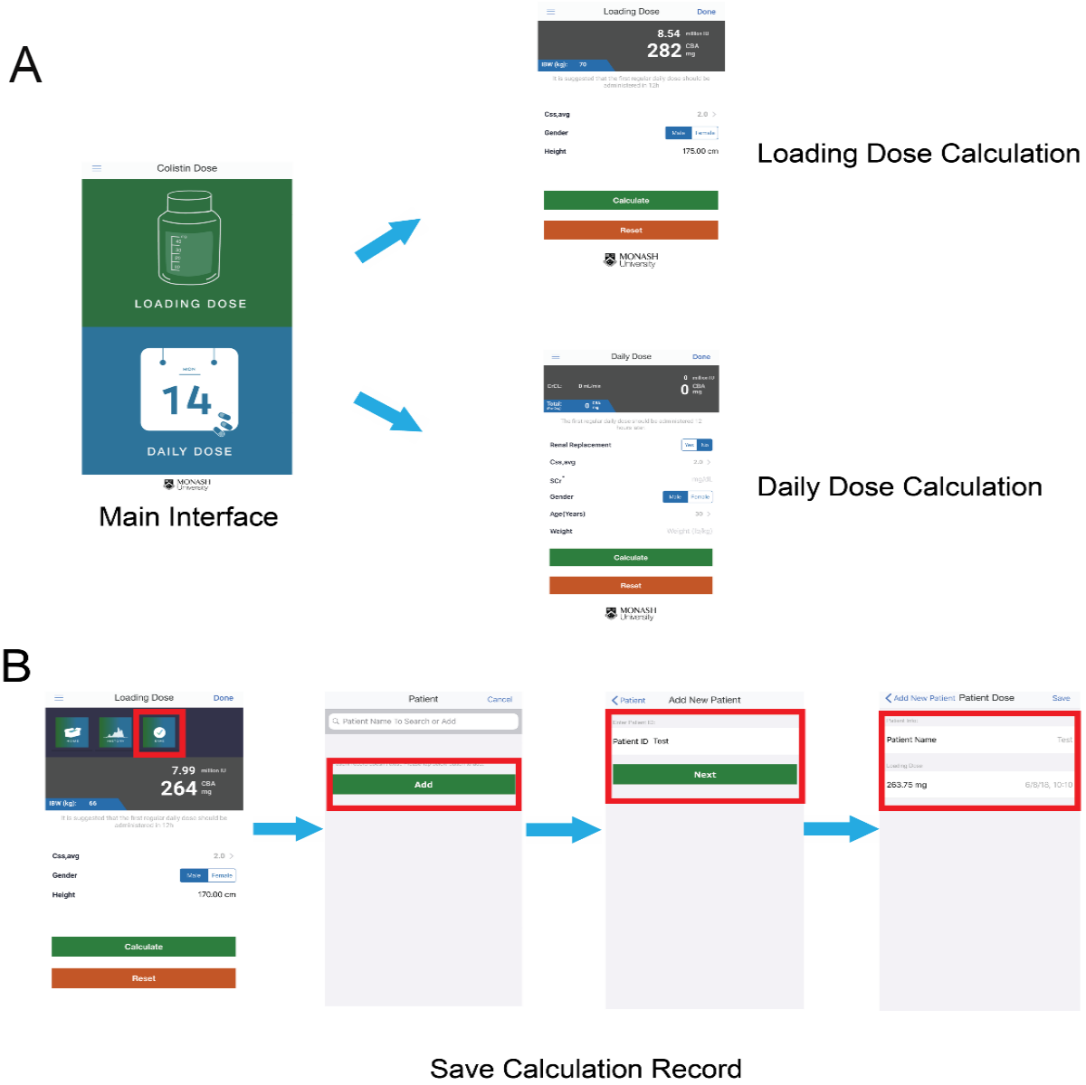
Screenshots illustrating the use of ColistinDose. (A) The main interface and calculations of the loading dose and daily dose. (B) Steps for saving the calculation results.

ColistinDose is simple to use and freely available for iOS (version 9 and above) at the Apple App Store. Upon opening the app to the main interface, the clinician is prompted to choose calculation of either a loading dose or a daily dose (Figure 2A). Prior to the publication of the final results of our population PK study [11], the importance of expediently achieving relatively high levels of formed colistin concentrations via a loading dose was already well known [10,11,17,18]. Following initiation of colistimethate treatment, the concentration of formed colistin increases slowly and may take up to 48 h before an acceptable average steady-state plasma concentration (C_ss,avg_) of formed colistin is achieved. Achieving therapeutic concentrations rapidly in critically ill patients is important, as suboptimal PK/PD and delayed effective therapy are associated with increased mortality rates [25]. Low concentrations may also lead to development of resistance [26]. In ColistinDose, calculation of the loading dose is based on three key parameters: (1) the targeted C_ss,avg_ of formed colistin, (2) gender (male/female), and (3) body height (inches or centimeters) (Figure 2A). Based on the recent population PK model, which found a colistin C_ss,avg_ of 2 mg/L to be a suitable initial target concentration for intravenous treatment of bloodstream infections and some minor infections when the colistin minimal inhibitory concentration (MIC) was ≤2 mg/L [11], the selectable C_ss,avg_ in ColistinDose has a default value of 2 mg/L and ranges from 0.5 mg/L to 4 mg/L, with a step of 0.5 mg/L. After pressing the “Calculate” button, the suggested loading dose is calculated using the following equation: CBA (mg) = C_ss,avg_ (mg/L) × 2.0 × IBW (kg), where the IBW is calculated using the Devine formula described in “Methods” [27]. It is important to note that should the recommended loading dose exceed the maximum recommended daily dose of 300 mg CBA (ie, 9 million IU) [23], a warning message (headed “Immediate Attention”) will pop up to alert the user that the loading dose has been capped at 300 mg CBA due to safety considerations. Importantly, to help clinicians from different parts of the world, the calculated dose is expressed in both CBA and million IU to avoid prescribing errors that have been known to occur due to confusion over the conversion [19].

Because colistimethate (the administered prodrug) is predominantly renally cleared [28], whereas the formed colistin is largely cleared by nonrenal pathways [29], the equations for the calculation of the daily (maintenance) dose are complicated and require information on the renal function of the patient and whether they are on RRT [11]. Once calculation of the daily dose is selected from the main interface of ColistinDose, the CrCl (mL/min) is determined via input of the patient’s serum creatinine (mg/dL), gender (male/female), age (years), and weight (lbs or kg). A screenshot of the daily dose calculation is shown in Figure 2A. The targeted C_ss,avg_ (mg/L) of formed colistin is also required [11]. Whether the patient is on RRT must also be selected. For patients not on RRT, the recommended daily dose is given in both CBA and million IU for 12-hourly administration (eg, 75 mg CBA per 12 h), with a suggestion that the first regular daily dose be administered 12 hours after the loading dose. This latter recommendation was taken from our population PK study [11]. For patients on RRT, three common dialysis options are provided: (1) IHD, (2) SLED, and (3) continuous RRT (CRRT). Based on our population PK analysis in patients receiving RRT, on a dialysis day of IHD, 20% or 50% of the baseline daily dose should be added after a 2-hour or 5-hour dialysis session, respectively [11]. For patients undergoing SLED or CRRT, 10% of the baseline daily dose should be supplemented for every hour of dialysis [11]. When the particular type of dialysis is chosen and a daily dose calculated, the user is reminded of this information with a screen prompt. For example, if CRRT is selected, the following message will appear below the dosing recommendations: “During CRRT, add 10% per 1 hour of CRRT to the baseline daily dose,” as suggested by our population PK study [11]. As per the loading dose, a warning message (headed “Immediate Attention”) pops up to alert the user whenever the calculated daily dose exceeds the maximum recommended 300 mg CBA (ie, 9 million IU) [11]. It should be noted that the actual calculated dose of colistimethate by ColistinDose can be higher, albeit with a cap of 300 mg.

Clinicians can choose to store the historical calculation records in the mobile device locally for each patient by using the “Save” option. Figure 2B demonstrates the saving of the dose calculations for a patient. With this function, clinicians can easily revisit previously calculated colistimethate doses as well as the types of renal dialysis, making dose adjustments where appropriate.

### Comparisons of Loading and Maintenance Daily Doses With Published Equations

To evaluate the accuracy of ColistinDose against our published dosing algorithms, we performed mock calculations of loading and maintenance daily doses using the height, age, and body weight of 8 “patients,” including 2 patients on each type of RRT (Multimedia Appendix 2). The calculated loading and daily doses for the 8 patients were almost identical to those calculated manually using the equations from the latest population PK model [11], the minor differences being due to number rounding. The rounded results are then converted to million IU. Overall, the results in Multimedia Appendix 2 demonstrate that the doses generated by ColistinDose accurately reflect doses recommended in patients with varying degrees of renal function and on different forms of RRT based on the most up-to-date published algorithms. In addition, coauthors JMP, IK, and KSK have evaluated and used ColistinDose in their clinical practices. The conversion function between two different dose definitions (ie, CBA versus IU) in ColistinDose provides a useful tool to prevent potential prescription errors.

## Discussion

### Comparison of ColistinDose With Existing Colistin Dosing Aids

Compared with the two existing dosing aids (ie, colistin dosing calculator [21] and colistin calculator [22]), the advantages of ColistinDose are twofold. First, ColistinDose utilizes equations derived from the most recent and comprehensive population PK model published in 2017 [11], whereas the colistin dosing calculator and colistin calculator utilize dosing algorithms based on an interim population PK analysis published in 2011 [10]. Prior to the publication of the latest population PK study [11], we had published an interim population PK analysis of intravenous colistimethate that examined 105 critically ill patients across 3 study sites (2 in the United States and 1 in Thailand), including 12 patients on hemodialysis and 4 patients on CRRT (3 on CVVHD and 1 on CVVH) [10]. A nonlinear mixed-effects modeling tool, S-ADAPT, was used to analyze plasma concentration-versus-time data for both colistimethate and colistin and found that their dispositions were best described by linear 2- and 1-compartment models, respectively. These models then formed the basis for the development of colistimethate dosing suggestions for various categories of patients based on the PK data and its integration with PD data for *Acinetobacter baumannii* and *Pseudomonas aeruginosa* in murine thigh and lung infection models [12]. Prior to the interim population PK analysis [10], information on the PK of colistimethate and formed colistin was scarce and had been reported in only 32 patients, all with CrCls >40 mL/min [18,30]. As previously discussed, while dosing recommendations for patients with a CrCl >30 mL/min were very similar between the interim and final population PK analyses, there were substantial differences for patients with lower CrCls, including patients on dialysis; in the final analysis, recommendations for these latter patients were approximately 100% higher than those suggested in the interim analysis. Use of the interim dosing equations thus risks exposing patients to subtherapeutic concentrations of the active component, colistin, decreasing the likelihood of efficacy and increasing the possibility of the emergence of colistin resistance. As colistimethate is a last-line therapy often used in critically ill patients when no other therapeutic options are available, attaining sufficient plasma concentrations is imperative given the potential consequences of treatment failure.

Second, a major advantage of ColistinDose is that once the app is downloaded and installed, all calculations are conducted on the local mobile device (iPhone or iPad) without the need for an internet connection. In contrast, the colistin dosing calculator [21] and colistin calculator [22] are only available online.

### Patients’ Privacy Protection

To protect the patient’s privacy and comply with ethics and data safety requirements, all data are only stored within and accessible from the app on the clinician’s mobile device; deleting or uninstalling the app will simultaneously delete all associated information permanently, as demonstrated in the disclaimer and terms of use issued by Monash University (refer to Multimedia Appendix 3). Clinicians are only required to input the patient’s ID—which could be either their patient ID number, a nickname, or anything easy to remember—to save the calculation results. Were the app to crash, only the crash data, not the patient’s information or previously saved calculation results, would be sent to the app developer via the iOS under the users’ agreement.

### Limitations and Outlook

The primary limitation of ColistinDose is that the user interface is in English. This may cause inconvenience for non–English-native clinicians to use the app. In light of this, we are considering adding multiple languages in the next version of ColistinDose. Because colistin has a narrow therapeutic window and different renal functions can cause substantial interpatient variability of the PK [31], therapeutic drug monitoring is highly recommended to ensure favorable clinical outcomes in patients. Incorporation of new therapeutic drug monitoring results will improve the precision of our population PK model, as well as the future version of ColistinDose.

### Conclusions

Colistimethate is increasingly used as a treatment of last resort in medically complex patients for infections caused by gram-negative “superbugs”. However, its complicated pharmacology and confusing dose definition can cause patient safety issues. Here, we have developed an easy-to-use mobile app, ColistinDose, that can be used at the bedside to facilitate the calculation of intravenous colistimethate dosage regimens for adult patients with varying degrees of renal function. To date, ColistinDose has been downloaded from 53 countries and regions around the globe. The potential for ColistinDose to improve patient care is significant, and its easy-to-use interface and functionalities can significantly assist clinicians worldwide to reduce prescribing errors, maximize efficacy, and minimize emergence of resistance and the likelihood of acute kidney injury in patients.

## Appendix

Multimedia Appendix 1 Detailed information of the open-source libraries and fonts utilized in ColistinDose.

Multimedia Appendix 2 Mock calculations of the maintenance daily doses in different patients.

Multimedia Appendix 3 Disclaimer and terms of use.

## Abbreviations

CBA: colistin base activity
CMS: colistin methanesulfonate
CrCl: creatinine clearance
CRRT: continuous renal replacement therapy
Css,avg: average steady-state plasma colistin concentration
CVVH: continuous veno-venous hemofiltration
CVVHD: continuous veno-venous hemodialysis
IBW: ideal body weight
IHD: intermittent hemodialysis
IU: international units
MIC: minimal inhibitory concentration
PD: pharmacodynamics
PK: pharmacokinetics
RRT: renal replacement therapy
SLED: sustained low-efficiency dialysis
