# Correction: ColistinDose, a Mobile App for Determining Intravenous Dosage Regimens of Colistimethate in Critically Ill Adult Patients: Clinician-Centered Design and Development Study

**DOI:** 10.2196/26593

**Published:** 2020-12-18

**Authors:** Xueliang Hua, Chen Li, Jason M Pogue, Varun S Sharma, Ilias Karaiskos, Keith S Kaye, Brian T Tsuji, Phillip J Bergen, Yan Zhu, Jiangning Song, Jian Li

**Affiliations:** 1 Independent Researcher Santa Clara, CA United States; 2 Department of Biochemistry and Molecular Biology Monash University Melbourne Australia; 3 Infection and Immunity Program Biomedicine Discovery Institute Monash University Melbourne Australia; 4 Institute of Molecular Systems Biology Department of Biology ETH Zurich Zurich Switzerland; 5 Department of Clinical Pharmacy University of Michigan College of Pharmacy Ann Arbor, MI United States; 6 1st Internal Medicine and Infectious Diseases Department Hygeia Hospital Marousi Greece; 7 Department of Internal Medicine University of Michigan Medical School Ann Arbor, MI United States; 8 Laboratory for Antimicrobial Dynamics NYS Center of Excellence in Bioinformatics & Life Sciences Buffalo, NY United States; 9 School of Pharmacy and Pharmaceutical Sciences University at Buffalo Buffalo, NY United States; 10 Department of Microbiology Monash University Melbourne Australia; 11 Monash Centre for Data Science Faculty of Information Technology Monash University Melbourne Australia

In “ColistinDose, a Mobile App for Determining Intravenous Dosage Regimens of Colistimethate in Critically Ill Adult Patients: Clinician-Centered Design and Development Study” (JMIR Mhealth Uhealth 2020;8(12):e20525) the authors noted one error due to an editing issue.

In the Abbreviations section, two abbreviations were listed incorrectly as follows:

CVVD: continuous veno-venous hemofiltration

CVVH: continuous veno-venous hemofiltration

These abbreviations have been corrected to:

CVVH: continuous veno-venous hemofiltration

CVVHD: continuous veno-venous hemodialysis

The correction will appear in the online version of the paper on the JMIR Publications website on December 18, 2020, together with the publication of this correction notice. Because this was made after submission to PubMed, PubMed Central, and other full-text repositories, the corrected article has also been resubmitted to those repositories.

