# Supplementary material for: ColistinDose, a Mobile App for Determining Intravenous Dosage Regimens of Colistimethate in Critically Ill Adult Patients: Clinician-Centered Design and Development Study
Source: JMIR Mhealth Uhealth. 2020 Dec 16;8(12):e20525. doi: 10.2196/20525 (PMC7748388; doi:10.2196/20525)
Supplement: Multimedia Appendix 1 [file mhealth_v8i12e20525_app1.docx]

**Multimedia Appendix 1**

**Table.** Detailed information of the opensource libraries and fonts utilized in *ColistinDose*.

| **Library** | **Opensource license/compliance with copyright** | **URL** |
| --- | --- | --- |
| Charts | Apache License version 2.0 | <https://github.com/danielgindi/Charts> |
| Former | The MIT License (MIT) | <https://github.com/ra1028/Former> |
| MGSwipeTableCell | The MIT License (MIT) | <https://github.com/MortimerGoro/MGSwipeTableCell> |
| Persei | The MIT License (MIT) | <https://github.com/Yalantis/Persei> |
| Realm | Apache License version 2.0 | <https://realm.io/> |
