# Supplementary material for: ColistinDose, a Mobile App for Determining Intravenous Dosage Regimens of Colistimethate in Critically Ill Adult Patients: Clinician-Centered Design and Development Study
Source: JMIR Mhealth Uhealth. 2020 Dec 16;8(12):e20525. doi: 10.2196/20525 (PMC7748388; doi:10.2196/20525)
Supplement: Multimedia Appendix 2 [file mhealth_v8i12e20525_app2.docx]

**Multimedia Appendix 2**

**Table.** Mock calculations of the maintenance daily doses in different patients.

| Patient | Patient 1 | Patient 2 | Patient 3 | Patient 4 | Patient 5 | Patient 6 | Patient 7 | Patient 8 |
| --- | --- | --- | --- | --- | --- | --- | --- | --- |
| Gender | Male | Female | Female | Male | Female | Male | Male | Female |
| Height (cm) | 175 | 158 | 175 | 158 | 175 | 158 | 175 | 158 |
| Age (year) | 43 | 68 | 43 | 68 | 43 | 68 | 43 | 68 |
| Body weight (kg) | 73 | 65 | 73 | 65 | 73 | 65 | 73 | 65 |
| RRT | Not on RRT | Not on RRT | IHD  (on dialysis day) | IHD  (on dialysis day) | SLED  (on dialysis day) | SLED  (on dialysis day) | CRRT  (on dialysis day) | CRRT  (on dialysis day) |
| Serum creatinine Concentration | 100 μM  (1.13 mg/dL) | 71 μM  (0.8 mg/dL) | - | - | - | - | - | - |
| Dialysis session (h) | - | - | 6 | 6 | 8 | 8 | 24 | 24 |
| Targeted C_ss,avg_ (mg/L) | 1 | 1.5 | 0.5 | 1 | 1.5 | 1 | 1.5 | 1 |
| Calculated *loading* dose using the appropriate formula in Nation *et al.*[11] | 140.9 mg CBA | 151.7 mg CBA | 66.0 mg CBA | 110.1 mg CBA | 197.9 mg CBA | 110.1 mg CBA | 211.4 mg CBA | 101.1 mg CBA |
| Calculated *loading* dose by *ColistinDose* | 141 mg CBA  (i.e. 4.27 MIU) | 152 mg CBA  (i.e. 4.60 MIU) | 66 mg CBA  (i.e. 2.00 MIU) | 110 mg CBA  (i.e. 3.34 MIU) | 198 mg CBA  (i.e. 6.00 MIU) | 110 mg CBA  (i.e. 3.34 MIU) | 211 mg CBA  (i.e. 6.41 MIU) | 101 mg CBA  (i.e. 3.06 MIU) |
| Calculated *daily* dose using the appropriate formula in Nation *et al*.[11] | 174.9 mg CBA | 215.1 mg CBA | 53.5 mg CBA | 106.9 mg CBA | 180.5 mg CBA | 120.3 mg CBA | 340.9 mg CBA | 227.2 mg CBA |
| Calculated *daily* dose by *ColistinDose* | 175 mg CBA  (i.e. 5.3 MIU) | 215 mg CBA  (i.e. 6.52 MIU) | 53 mg CBA  (i.e. Baseline 33 mg CBA [1.01 MIU] + Supplement after 6 h: 20 mg CBA [0.61 MIU]) | 107 mg CBA  (i.e. Baseline 67 mg CBA [2.03 MIU] + Supplement after 6 h: 40 mg CBA [1.22 MIU]) | 180 mg CBA  (i.e. Baseline 100 mg CBA [3.04 MIU] + Supplement per hour × 8 h: 10 mg CBA [0.30 MIU]) | 123 mg CBA  (i.e. Baseline 67 mg CBA [2.03 MIU] + Supplement per hour × 8 h: 7 mg CBA [0.20 MIU]) | 340 mg CBA  (i.e. Baseline: 100 mg CBA [3.04 MIU] + Supplement per hour × 24 h: 10 mg CBA [0.3 MIU])* | 235 mg CBA  (i.e. Baseline: 67 mg CBA [2.03 MIU] + Supplement per hour × 24 h: 7 mg CBA [0.20 MIU]) |

*With this dose calculation in *ColistinDose* the following warning is also provided: **Immediate Attention** – The calculated dose (340 mg CBA) is >300 mg CBA.

IHD: intermittent hemodialysis.

SLED: sustained low-efficiency dialysis.

CRRT: continuous renal replacement therapy.

RRT: renal replacement therapy.

C_ss,avg_: average steady-state plasma concentration.

CBA: colistin base activity.

MIU: million international units.
