# Supplementary material for: ColistinDose, a Mobile App for Determining Intravenous Dosage Regimens of Colistimethate in Critically Ill Adult Patients: Clinician-Centered Design and Development Study
Source: JMIR Mhealth Uhealth. 2020 Dec 16;8(12):e20525. doi: 10.2196/20525 (PMC7748388; doi:10.2196/20525)
Supplement: Multimedia Appendix 3 [file mhealth_v8i12e20525_app3.docx]

**Multimedia Appendix 3**

**Disclaimer and Terms of Use.**

Any and all dosing information generated through the use of ColistinDose is indicative and provided for use by medical practitioners for information, education and research purposes only. USERS MUST CONFIRM ALL CALCULATIONS AND EXERCISE THEIR OWN INDEPENDENT CLINICAL JUDGMENT WHEN USING COLISTINDOSE.

Monash University makes no warranties or representations of any kind, express or implied, including but not limited to, implied warranties of title or non-infringement of intellectual property, or accuracy, reliability, completeness or appropriateness of the use of ColistinDose for the calculation of loading and daily doses of intravenous colistimethate. The algorithms employed in ColistinDose for the calculation of both loading and daily doses of intravenous colistimethate are based on the population pharmacokinetic model (*Clin Infect Dis.* 2017. 64(5):565-571). However, Monash University reserves the right to make changes to ColistinDose at any time without notice and it remains the responsibility of the user to keep up to date with any development or change in loading or dosage recommendations. To the extent permitted by law, all liability for any injury, death, loss or any direct, indirect, incidental, consequential, special, punitive or other damages whatsoever, in connection with the use of ColistinDose in any manner, is hereby excluded.

ColistinDose will not store any personal or identity information unless a user chooses to save the calculation record to the user’s mobile device only (optional). Where this occurs, it is the responsibility of the user to ensure that any and all information saved to the device is stored and accessed in a manner which complies with all relevant laws and health provider policies concerning the collection, use, storage and disclosure of health records and personal information.

Copyright ^©^ Monash University 2018 and Xueliang Hua. All rights reserved. Except as provided in the Copyright Act 1968, this work may not be used, reproduced, adapted or communicated without the written consent of the University.
